# Effects of isoflurane and urethane anesthetics on glutamate neurotransmission in rat brain using in vivo amperometry

**DOI:** 10.1186/s12868-023-00822-3

**Published:** 2023-10-10

**Authors:** Joshua A. Beitchman, Gokul Krishna, Caitlin E. Bromberg, Theresa Currier Thomas

**Affiliations:** 1 Department of Child Health, University of Arizona College of Medicine - Phoenix, 425 N. 5th St. | 322 ABC-1 Building, Phoenix, AZ 85004-2127 USA; 2 Barrow Neurological Institute at Phoenix Children’s Hospital, Phoenix Children’s Hospital, Phoenix, AZ USA; 3 College of Graduate Studies, Midwestern University, Glendale, AZ USA; 4 Phoenix VA Healthcare System, Phoenix, AZ USA

**Keywords:** Isoflurane, Urethane, Glutamate neurotransmission, Amperometry, Cortex, Hippocampus, Thalamus

## Abstract

**Background:**

Aspects of glutamate neurotransmission implicated in normal and pathological conditions are predominantly evaluated using in vivo recording paradigms in rats anesthetized with isoflurane or urethane. Urethane and isoflurane anesthesia influence glutamate neurotransmission through different mechanisms; however, real-time outcome measures of potassium chloride (KCl)-evoked glutamate overflow and glutamate clearance kinetics have not been compared within and between regions of the brain. In order to maintain rigor and reproducibility within the literature between the two most common methods of anesthetized in vivo recording of glutamate, we compared glutamate signaling as a function of anesthesia and brain region in the rat strain most used in neuroscience.

**Methods:**

In the following experiments, in vivo amperometric recordings of KCl-evoked glutamate overflow and glutamate clearance kinetics (uptake rate and T_80_) in the cortex, hippocampus, and thalamus were performed using glutamate-selective microelectrode arrays (MEAs) in young adult male, Sprague-Dawley rats anesthetized with either isoflurane or urethane.

**Results:**

Potassium chloride (KCl)-evoked glutamate overflow was similar under urethane and isoflurane anesthesia in all brain regions studied. Analysis of glutamate clearance determined that the uptake rate was significantly faster (53.2%, p < 0.05) within the thalamus under urethane compared to isoflurane, but no differences were measured in the cortex or hippocampus. Under urethane, glutamate clearance parameters were region-dependent, with significantly faster glutamate clearance in the thalamus compared to the cortex but not the hippocampus (p < 0.05). No region-dependent differences were measured for glutamate overflow using isoflurane.

**Conclusions:**

These data support that amperometric recordings of KCl-evoked glutamate under isoflurane and urethane anesthesia result in similar and comparable data. However, certain parameters of glutamate clearance can vary based on choice of anesthesia and brain region. In these circumstances, special considerations are needed when comparing previous literature and planning future experiments.

## Highlights

- Potassium-evoked glutamate overflow was similar under urethane and isoflurane anesthesia.
- Glutamate clearance parameters were similar under both anesthesias in the cortex and hippocampus but not the thalamus.
- Glutamate clearance kinetics differ between brain regions when anesthetized with urethane.
- Experimental design, brain region of interest, and outcome parameters of glutamate clearance should be considered when designing anesthetized amperometry recordings of glutamate.

## Introduction

Amperometric techniques have provided fundamental information regarding brain chemical communication of neurotransmitter systems in normal and disease-related physiologies. Glutamate is the primary excitatory neurotransmitter in the central nervous system (CNS) [1]. Experimental models of many CNS disorders, including Alzheimer’s disease (AD), Huntington’s disease (HD), Parkinson’s disease (PD), epilepsy, attention-deficit/hyperactivity disorder (ADHD) and post-concussive symptoms have indicated changes in glutamate neurotransmission as part of their pathophysiology [2–6]. Importantly, mechanisms controlling glutamate neurotransmission provide novel targets for pharmacological intervention to restore physiological norms.

**Fig. 1:**
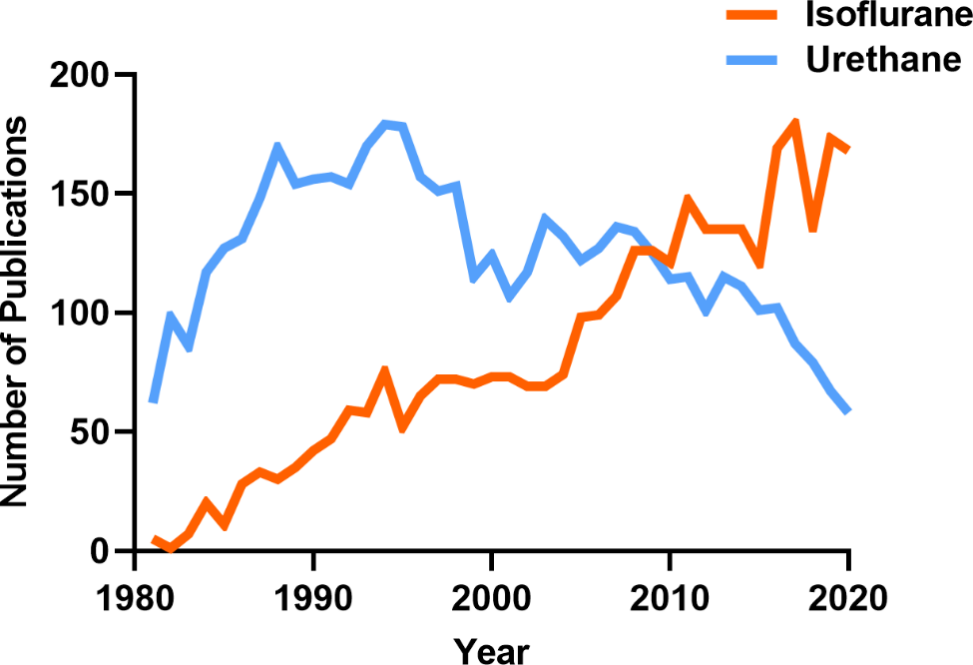
**The number of publications using urethane and isoflurane for neuroscience research between 1981–2020**. A PubMed search indicates the use of urethane has decreased while the use of isoflurane has increased over the past 40 years

Laboratory studies often use different anesthetics when evaluating functional alterations in neurocircuitry. These anesthetics can influence glutamate neurotransmission through interactions with various molecular targets making it challenging to determine if observed differences are due to pathophysiology or anesthetic used. Thus, meta-analysis and literature reviews that do not consider anesthetic choice may result in inaccurate interpretations given the lack of information on the influence that anesthesia has on certain outcome measures of glutamate kinetics. Despite the widespread use of various anesthetics, the fundamental question remains whether different anesthetics can influence neurochemical signaling and their mechanism of action. Urethane and isoflurane are commonly used anesthetics in translational neuroscience experiments, with ~ 8290 papers identified for urethane and isoflurane since 1981 (PubMed search for “urethane” AND “brain” or “isoflurane” AND “brain” from 1981 to 2020; Fig. 1). In 1981, only five publications used isoflurane compared to 62 using urethane (7.5% isoflurane use). In 2020, 117 publications used isoflurane, and only 59 used urethane (66.5% isoflurane use). With this transition, it is important to consider whether specific outcome measures of experiments are influenced differentially between the two anesthetics so that researchers can build on previous bodies of knowledge. Furthermore, this knowledge would be a necessary consideration for experimental design and interpretation of future studies evaluating aspects of neuronal glutamatergic communication as we continue to apply them to understand neurochemical signaling under homeostatic and pathological conditions.

Potassium (KCl)-evoked glutamate overflow and glutamate clearance kinetics are commonly used metrics of glutamatergic communication, measured by microdialysis or amperometry in anesthetized rats. Both urethane and isoflurane are known to suppress glutamate neurotransmission but work through dissimilar mechanisms that could differentially influence glutamate signaling. Urethane, also known as ethyl carbamate, produces long-lasting, steady levels of anesthesia with reports indicating minimal effects on circulation, respiration, autonomic function, and GABA neurotransmission [7–11]. Recent complementary studies indicate that urethane anesthetized rats retained functional connectivity patterns most similar to awake animals in comparison to isoflurane [11]. While urethane remains a popular anesthetic for electrophysiological experiments, but the actual influence on evoked glutamate release and clearance is unknown.

Given the benefits and ease of urethane use, urethane’s reported carcinogenic properties primarily constrain urethane use to non-survival experiments [12, 13]. To use a more clinically relevant anesthetic, an increasing number of research papers using electrochemical and electrophysiological techniques have begun to use isoflurane. Isoflurane, a halogenated ether, has been implemented for its swift induction and recovery times time [14]. The link between isoflurane exposure and synaptic transmission has been supported with studies showing isoflurane inhibition of voltage-gated sodium currents contributing to suppression of glutamate release in prefrontal cortex [15]. Recent ex vivo evidence also suggests that neonatal isoflurane exposure reduced glutamate uptake in cortical slices [16]. Isoflurane is also shown to enhance GABAergic neurotransmission in a dose-dependent fashion [17], where increased inhibition may result in decreased amounts of KCl-evoked glutamate release or may be similar to urethane due to full anesthetic dose.

In this study, we sought to evaluate the relative comparability of KCl-evoked glutamate overflow and glutamate clearance kinetics collected via amperometric recordings from rats anesthetized by either isoflurane or urethane. Glutamate selective microelectrode arrays coupled with micropipettes filled with isotonic KCl solution or exogenous glutamate were placed within the cortex, hippocampus, or thalamus of naïve rats anesthetized with either isoflurane or urethane. The target regions were chosen because they are commonly implicated in cognition, somatosensation, neuropathic pain, and alterations in circuit function.

## Materials and methods

### Subjects

A total of 24 young adult male Sprague-Dawley rats (3–4 month old; 359–438 g) were purchased (Envigo, Indianapolis, IN) and pair housed in disposable cages (Innovive, San Diego, CA) under normal 12:12 h light:dark cycle in a temperature- and humidity-controlled vivarium. Rats were provided food (Teklad 2918, Envigo, Indianapolis, IN) and water *ad libitum.* Group size estimates were determined from previous work [18], where *n* = 6–10/group could achieve > 80% power to detect a 75% increase in KCl-evoked glutamate release. All procedures were conducted in accordance with the National Institutes of Health (NIH) Guidelines for the Care and Use of Laboratory Animals care and were approved by the University of Arizona College of Medicine-Phoenix Institutional Animal Care and Use Committee (protocol #18–384).

### Microelectrode arrays

Ceramic-based MEAs encompassing four platinum recording surfaces (15 × 333 μm; S2 configuration) aligned in a dual, paired design were obtained from Quanteon LLC (Nicholasville, KY) for in vivo anesthetized recordings. MEAs were fabricated and selected for recordings using previously described measures of 0.125% glutaraldehyde and 1% GluOx [18–22]. MEAs were made glutamate selective as previously described [18, 23, 24]. Prior to in vivo recordings, a size exclusion layer of m-phenylenediamine dihydrochloride (Acros Organics, NJ) was electroplated to all four platinum recording sites with the use of the FAST16 mkIII system (Fast Analytical Sensor Technology Mark III, Quanteon, LLC, Nicholasville, KY) to block potential interfering analytes such as ascorbic acid, catecholamines and other indoleamines [25]. The four platinum recording sites consisted of two glutamate-sensitive and two sentinel channels. The glutamate-sensitive channels were coated with 1% BSA, 0.125% glutaraldehyde and 1% glutamate oxidase.

### Microelectrode array calibration

**Fig. 2:**
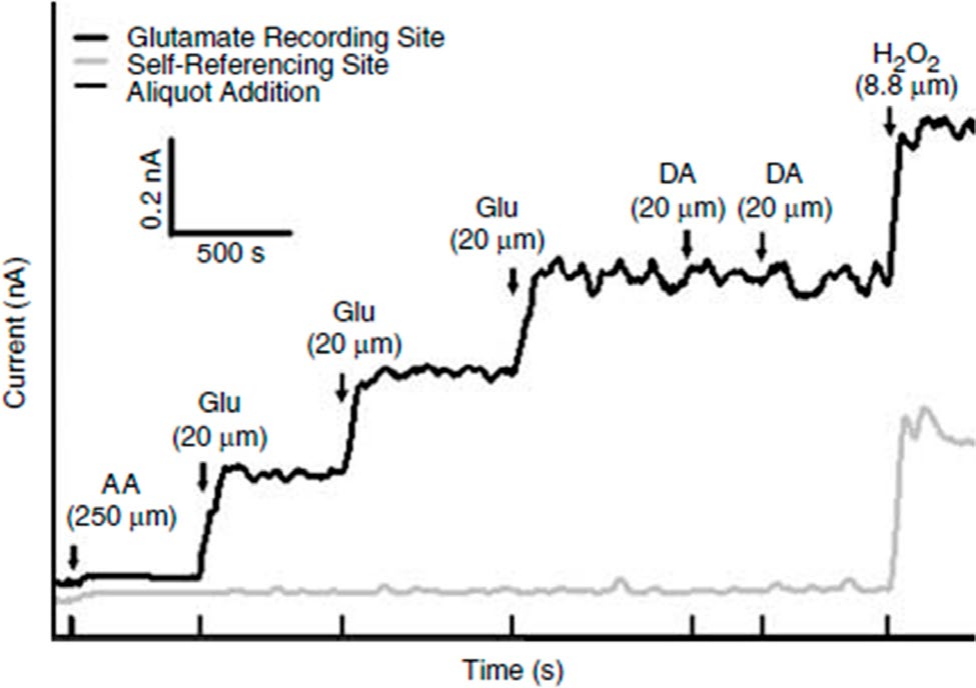
**Representative calibration of a glutamate selective microelectrode array (MEA)**. Representative MEA calibration prior to in vivo recordings in which only one glutamate selective recording site and one self-referencing site are represented. Aliquots of 250 µM ascorbic acid (AA), 20 µM glutamate (Glu), 2 µM dopamine (DA), and 8.8 µM H_2_O_2_ are represented by vertical bars on the x-axis

MEAs were calibrated with a FAST16 mkIII system to accurately record glutamate concentrations by creating a standard curve for each coated MEA before in vivo recordings, as described previously [23]. Briefly, the MEA tips were submerged into 40 ml of stirred 0.05 M phosphate-buffered saline (PBS) solution, kept in a water bath, and allowed to reach a stable baseline before beginning the calibration. Aliquots from stock solutions were added in succession following equilibrium such that 500 µL of ascorbic acid, three additions of 40 µL of L-glutamate, two additions of 20 µL of dopamine, and 40 µL of H_2_O_2_ were added to produce final concentrations of 250 µM of ascorbic acid, 20, 40 and 60 µM of L-glutamate, 20 µM and 40 µM dopamine and 8.8 µM of H_2_O_2_ respectively in the beaker of PBS (pH 7.4). A representative calibration is depicted in Fig. 2. From the MEA calibration, the following metrics were calculated and used for the determination of inclusion within the in vivo recordings: slope (sensitivity to glutamate), the limit of detection (LOD) (lowest amount of glutamate to be reliably recorded), and selectivity (ratio of glutamate to ascorbic acid). For the study, a total of 48 MEAs were used with 96 recording sites (Slopes: >5 pA/µM, LOD: <2.5 µM, and selectivity: >20:1).

### Microelectrode array/micropipette assembly

Following calibration, a single-barrel glass micropipette was attached to the MEA with the following steps to allow for the local application of solutions during in vivo experiments. A single-barreled glass capillary with a filament (1.0 × 0.58 mm^2^, 6” A-M Systems, Inc., WA) was pulled to a tip using a Kopf Pipette Puller (David Kopf Instruments, CA). The pulled pipette was then bumped using a microscope and a glass rod to have an inner diameter averaging 11.1 µm ± 0.56. The pulled glass pipettes were embedded in modeling clay and attached to the circuit board above the MEA tip. Molten wax was applied to the embedded pipette to secure the MEA/micropipette assembly and prevent its movement during the recording. The pipette attachment was performed under a microscope to carefully place the tip of the pipette above the glutamate-sensitive sites from the surface of the electrode. Measurements of pipette placement were confirmed using a microscope with a calibrated reticule in which the pipette was approximately 45 to 105 µm from the electrode sites (averaging 78.7 µm ± 4.9).

### Reference electrode assembly

Silver/silver chloride reference electrodes were fabricated from Teflon-coated silver wire to provide an in vivo reference for the MEA. A 0.110-inch Teflon-coated silver wire (A-M Systems, Carlsborg, WA) was prepared by stripping approximately ¼” of Teflon coating from each end of a 6” section, soldering one end to a gold-plated socket (Ginder Scientific, Ottawa, ON) and the other being prepared to be coated with silver chloride. This end was placed into a 1 M HCl saturated with NaCl plating solution. A 9 V current was applied to the silver wire (cathode) versus the platinum wire (anode) for approximately 5 min. Upon completion, the silver/silver chloride reference electrode was placed in a light-sensitive box until implanted.

### Surgery

Rats were randomly assigned to receive either isoflurane or urethane. For isoflurane anesthesia, rats were initially anesthetized with 5% isoflurane (in 100% oxygen at a flow rate of 0.5 l/min) for 5 min in an induction chamber. After righting and pedal reflexes were lost, hair was shaved from the head, and the animal was secured in a stereotaxic frame (David Kopf Instruments) with nonterminal ear bars. The frame was equipped with a nose cone for continuous delivery of 2% isoflurane using a calibrated vaporizer for the remainder of the experiment. 25% urethane (Sigma Aldrich, St. Louis, MO) was administered as serial intraperitoneal (i.p.) injections. After the initial dose of 1 g/kg urethane, supplementary doses of 0.1–0.2 g/kg (i.p.) were administered every 20 min until the pedal reflex was lost, a total dose between 1.25 and 1.5 g/kg. Heads were shaved and each rat was placed into a stereotaxic apparatus. Body temperature was maintained at 37 °C with isothermal heating pads (Braintree Scientific, MA). After the betadine/alcohol wash and allowed to dry, a midline incision was made in which the skin, fascia, and temporal muscles were reflected, exposing the skull. A bilateral craniectomy was then performed using a Dremel, exposing the stereotaxic coordinates for the somatosensory cortex, hippocampus, or thalamus. Dura was removed prior to the implantation of the MEA. Brain tissue was hydrated by applying saline-soaked cotton balls and gauze. Using blunt dissection, a silver/silver chloride-coated reference electrode wire was placed in a subcutaneous pocket on the dorsal side of the rat [26, 27]. All experiments were performed during the light phase of the 12 h-dark/light cycle.

### In vivo amperometric recordings

Amperometric recordings performed here under either urethane or isoflurane anesthesia were similar to previously published methods [18, 28, 29]. For recording procedures with both anesthetics, body temperature was maintained at 37 °C with a heating pad and the level of anesthesia depth was assessed throughout the experiment to ensure an adequate level of anesthesia by continuously monitoring pedal reflex, muscle relaxation, eyelid reflex, and appearance of slower and more rhythmic breathing.

Immediately prior to implantation of the glutamate-selective MEA-pipette assembly, the pipette was filled with isotonic 120 mM of KCl (120 mM KCl, 29 mM NaCl, 2.5 mM CaCl_2_, pH 7.2 to 7.5) or 100 µM L-glutamate (100 µM L-glutamate in 0.9% sterile saline pH 7.2–7.5). The concentrations for both solutions have been previously shown to elicit reproducible potassium-evoked glutamate overflow or exogenous glutamate peaks [20, 30, 31]. Solutions were filtered through a 0.20 μm sterile syringe filter (Sarstedt AG & Co. Numbrecht, Germany) attached to a 1 mL syringe with a 4-inch, 30-gauge stainless steel needle with a beveled tip (Popper and Son, Inc, NY) while filling the micropipette. The open end of the micropipette end was then connected to a Picospritzer III (Parker-Hannin Corp., General Valve Corporation, OH) with settings to dispense fluid through the use of nitrogen gas in nanoliter quantities as measured by a dissecting microscope (Meiji Techno, San Jose, CA) with a calibrated reticule in the eyepiece [32, 33].

**Fig. 3:**
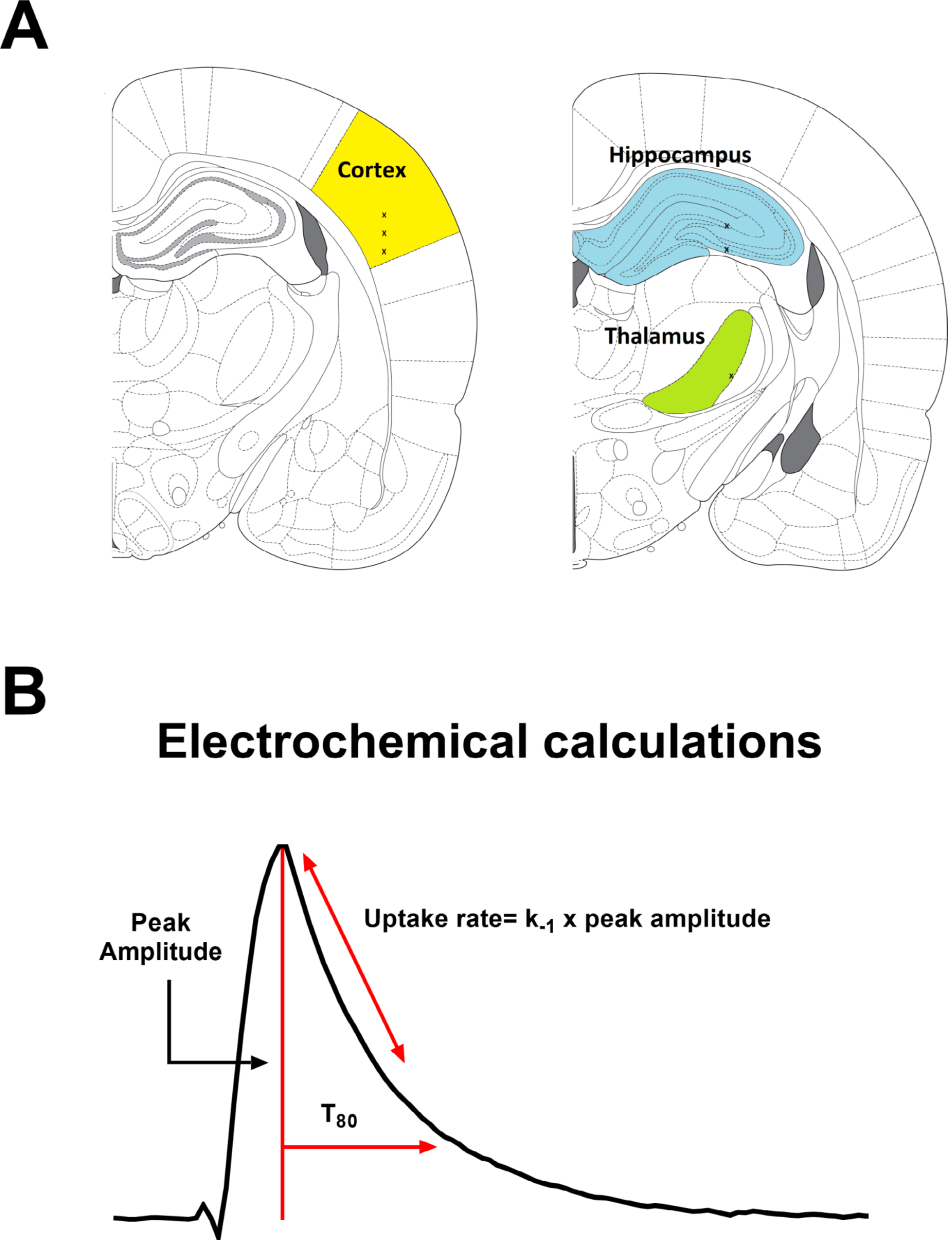
**Recording regions and amperometric calculations**. (**A**) Anatomical regions of interest (ROI) in the rodent brain were the hippocampus (blue), thalamus (green), and cortex (yellow). Image modified from Paxinos and Watson (2007). * represents the tip of the MEA. (**B**) Representative peak, showing glutamate concentration (µM) as a function of time in seconds in response to local applications of 100 µM glutamate. Amperometric calculations used in the analysis (peak amplitude, uptake rate, and T_80_) are shown

Once the MEA-micropipette apparatus was securely attached to the Picospritzer and FAST system, bregma was measured using an ultraprecise stereotaxic arm. MEA-micropipette constructs were implanted in the cortex (AP, -2.8 mm; ML, ± 5.0 mm; DV, -1.0 mm vs. bregma), hippocampus (AP, -3.5 mm; ML, ± 3.0 mm; DV, -2.6 to -3.75 mm vs. bregma) and thalamus (AP, -3.5 mm; ML, ± 3.0 mm; DV, -5.6 mm vs. bregma) based on the coordinates from Paxinos and Watson [34] (Fig. 3A). A constant voltage was applied to the MEA using the FAST16 mkIII recording system. In vivo recordings were performed at an applied potential of + 0.7 V compared to the silver/silver chloride reference electrode wire. All data were recorded at a frequency of 10 Hz and amplified by the headstage piece (2 pA/mV). Glutamate and KCl-evoked measures were recorded in both hemispheres in a randomized and balanced experimental design to mitigate possible hemispheric variations or effects of anesthesia duration.

### KCl-Evoked overflow of glutamate analysis parameters

All recordings were conducted at 10 Hz and analyzed without signal processing or filtering the data. Once the glutamate MEA was lowered, and the electrochemical signal had reached stable baseline, matched volumes of 120 mM potassium were locally applied two minutes apart to induce depolarization and subsequent overflow of glutamate from synapse was measured by the MEA. The volume of each application of KCl was predetermined to ensure maximal response (largest amount of glutamate released), which was confirmed by a smaller evoked peak following the 2-minute interval. Volume of locally applied exogenous fluids are determined using a stereomicroscope fitted with reticule and calibrated for 250 nL release per 1 mm movement of the meniscus within the single barrel glass micropipette [33, 35]. The pressure injections usually lasted for 1 s at 20 psi. The maximal amplitude of the glutamate response (µM) of the first peak was the primary outcome measure for analysis.

### Glutamate clearance analysis parameters

All recordings were conducted at 10 Hz and analyzed without signal processing or filtering of the data. Once the electrochemical signal had reached a baseline, 100 µM glutamate was locally applied into the extracellular space to record glutamate detection and subsequent clearance. Additions of exogenous glutamate were applied at 30-second intervals for reproducible glutamate peaks detected by MEAs where signals returned to the baseline before repeating the procedure. Clearance parameters of 3 amplitude-matched peaks were averaged to create a single representative value per recorded region per rat. Primary outcome measures for analysis include the uptake rate and the time taken for 80% of the exogenous glutamate to clear the extracellular space (T_80_). The uptake rate is calculated by multiplying the uptake rate constant (k_− 1_) by the peak’s maximum amplitude. The uptake rate constant is how quickly the glutamate decays over time and is calculated by fitting a linear regression to the natural log transformation of the data over time [35]. Diagrammatic example of these calculations is shown in Fig. 3B.

### MEA placement verification

Immediately following in vivo recordings, isoflurane-anesthetized rats received lethal injection (4 ml/kg, i.p.) of Euthasol® euthanasia solution (sodium pentobarbital and phenytoin mixture, Virbac AH, Inc.). Upon cessation of breathing, rats were decapitated, brains excised, and post-fixed in cold 4% paraformaldehyde (PFA). Rats anesthetized with urethane were decapitated, brains excised, and placed in 4% PFA. Brains were cryoprotected, sectioned, and stained with a hematoxylin and eosin stain to confirm MEA placement. No electrode tracts were excluded due to placement (data not shown).

### Statistical analysis

The amperometric data were saved on the FAST 16 mkIII system. Datasets were analyzed with FAST Analysis software (Jason Burmeister Consulting) and processed using a customized Microsoft Excel® spreadsheet. Inclusion criterion for data analysis of KCl-evoked response was the maximum amount of glutamate overflow. When evaluating glutamate clearance kinetics, only amplitude values between 10 and 23 µM were considered, accounting for the influence of Michaelis-Menten kinetics. This selection criteria stems from the understanding that as glutamate concentration rises, the transporters’ reaction rate, and consequently the glutamate clearance rate, also increases [36]. All data are presented as mean + standard error mean (mean + SEM) and analyzed using the statistical software GraphPad Prism 9.4.1. The number of animals comparisons between anesthetics used a two-tailed Student’s *t*-test. Differences between regions were determined using a one-way ANOVA with Tukey’s post-hoc comparison. All data sets were evaluated for variance (Brown-Forsythe) and normality (Kolmogorov-Smirnov) to ensure assumptions were met for analysis. Outliers were evaluated using a ROUT method with a false discovery rate of (Q) 1% (GraphPad Prism Version 9.5.1, San Diego, CA). Differences were considered statistically significant when p < 0.05.

## Results

### Levels of evoked release of glutamate were similar in urethane and isoflurane-anesthetized rats

**Fig. 4:**
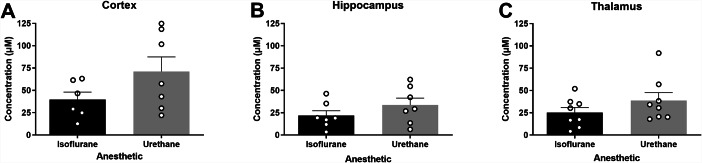
**Levels of evoked release of glutamate were similar in urethane and isoflurane-anesthetized rats**. Local applications of volume-matched 120 mM potassium chloride (KCl) were made to the cortex, hippocampus, and thalamus. There was no significant difference between the glutamate overflow measured for rats anesthetized with isoflurane or urethane in the (**A**) cortex (*t*_11_ = 1.629, p = 0.131), (**B**) hippocampus (*t*_12_ = 1.247, p = 0.236), or (**C**) thalamus (*t*_14_ = 1.299, p = 0.214). Bar graphs represent mean + SEM. *N* = 6–8 per group

Surrounding neuronal tissue was depolarized with volume-matched (75–150 nL) applications of 120 mM isotonic KCl, and the maximum amplitude of glutamate was recorded. Cortical recordings revealed no significant differences in the amount of glutamate overflow between rats anesthetized with isoflurane or urethane (*N* = 6–7 rats; $\stackrel{-}{\text{x}}$_Iso_ = 39.65 µM, $\stackrel{-}{\text{x}}$_Ure_ = 71.14 µM; *t*_11_ = 1.629; p = 0.131; Fig. 4A). Similarly, KCl-evoked glutamate overflow was independent of anesthetics in the hippocampus (*N* = 7 rats; $\stackrel{-}{\text{x}}$_Iso_ = 21.80 µM, $\stackrel{-}{\text{x}}$_Ure_ = 33.66 µM; *t*_12_ = 1.247; p = 0.236; Fig. 4B) or thalamus (*N* = 8 rats; $\stackrel{-}{\text{x}}$_Iso_ = 25.18 µM, $\stackrel{-}{\text{x}}$_Ure_ = 38.84 µM; *t*_14_ = 1.299; p = 0.214; Fig. 4C).

### Glutamate uptake rate was faster in the thalamus of urethane-anesthetized rats

**Fig. 5:**
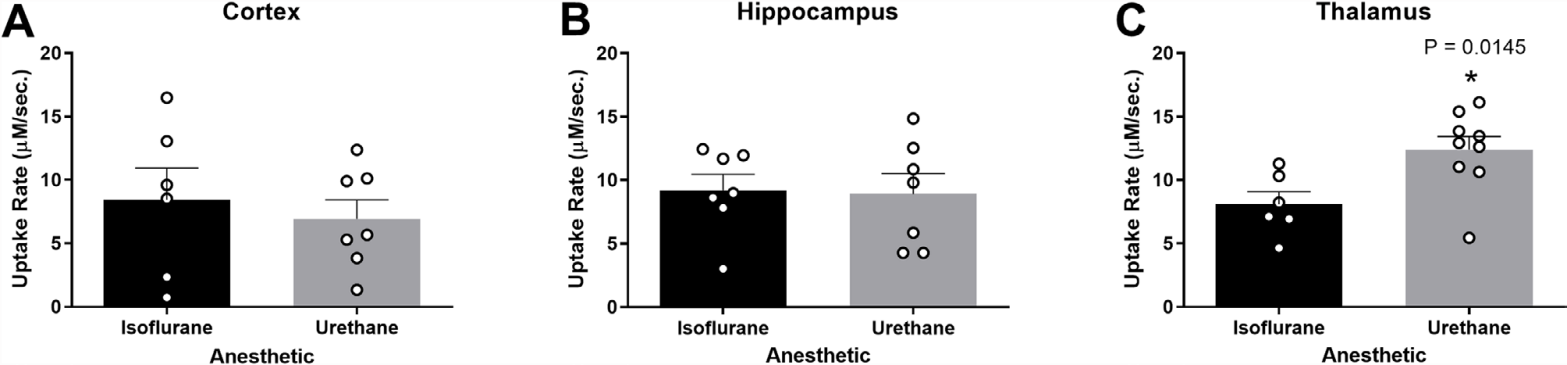
**Glutamate uptake rates were similar in the cortex and hippocampus and different in the thalamus of urethane and isoflurane-anesthetized rats**. Amplitude-matched signals from local applications of 100 µM exogenous glutamate compared for extracellular glutamate clearance in the cortex, hippocampus, and thalamus. No significant difference in the uptake rate between rats anesthetized with isoflurane or urethane in the (**A**) cortex (*t*_11_ = 0.544, p = 0.597) and (**B**) hippocampus (*t*_12_ = 0.148, p = 0.884). (**C**) Urethane administration was associated with a significantly faster uptake rate in thalamus than isoflurane (*t*_13_ = 2.817, p = 0.0145). Bar graphs represent mean + SEM. *N* = 6–9 per group

**Fig. 6:**
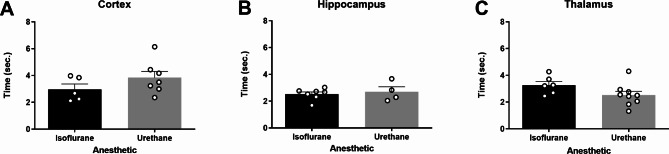
**Extracellular clearance times (T**_**80**_**) were similar in urethane and isoflurane-anesthetized rats.** Local application of 100 µM exogenous glutamate resulted in amplitude-matched signals to assess the T_80_ in the cortex, hippocampus, and thalamus. There was no significant difference in the time taken for 80% of the maximal amplitude to clear between rats anesthetized with isoflurane or urethane in the (**A**) cortex (*t*_10_ = 1.329, p = 0.213), (**B**) hippocampus (*t*_9_ = 0.525, p = 0.612), or (**C**) thalamus (*t*_13_ = 1.827, p = 0.090). Bar graphs represent mean + SEM. *N* = 4–9 per group

In each region of interest (ROI), glutamate clearance from the extracellular space was evaluated. Local applications of 100 µM exogenous glutamate were amplitude-matched at the time of administration. Cortical recordings revealed no significant differences in uptake rate between rats anesthetized with isoflurane or urethane (*N* = 6–7 rats; $\stackrel{-}{\text{x}}$_Iso_ = 8.460 µM/sec., $\stackrel{-}{\text{x}}$_Ure_ = 6.938 µM/sec.; *t*_11_ = 0.544; p = 0.597; Fig. 5A). Uptake rate was also similar between anesthetics in the hippocampus (*N* = 7 rats; $\stackrel{-}{\text{x}}$_Iso_ = 9.221 µM/sec., $\stackrel{-}{\text{x}}$_Ure_ = 8.923 µM/sec.; *t*_12_ = 0.148; p = 0.884; Fig. 5B). However, the thalamus of urethane-anesthetized rats showed a significantly faster uptake rate in comparison to isoflurane-anesthetized rats (*N* = 6–9 rats; $\stackrel{-}{\text{x}}$_Iso_ = 8.093 ± 1.467 µM/sec., $\stackrel{-}{\text{x}}$_Ure_ = 12.40 ± 1.558 µM/sec.; *t*_13_ = 2.817; p = 0.0145; Fig. 5C). No differences were identified in the cortex (*N* = 5–7 rats; $\stackrel{-}{\text{x}}$_Iso_ = 2.972 s, $\stackrel{-}{\text{x}}$_Ure_ = 3.834 s; *t*_10_ = 1.329; p = 0.213; Fig. 6A), hippocampus (*N* = 5–7 rats; $\stackrel{-}{\text{x}}$_Iso_ = 2.529 s, $\stackrel{-}{\text{x}}$_Ure_ = 2.708 s; *t*_9_ = 0.525; p = 0.612; Fig. 6B), or thalamus (*N* = 6–9 rats; $\stackrel{-}{\text{x}}$_Iso_ = 3.269 s, $\stackrel{-}{\text{x}}$_Ure_ = 2.525 s; *t*_13_ = 1.827; p = 0.090; Fig. 6C).

### Urethane anesthesia causes brain region-specific differences in glutamate kinetics

**Fig. 7:**
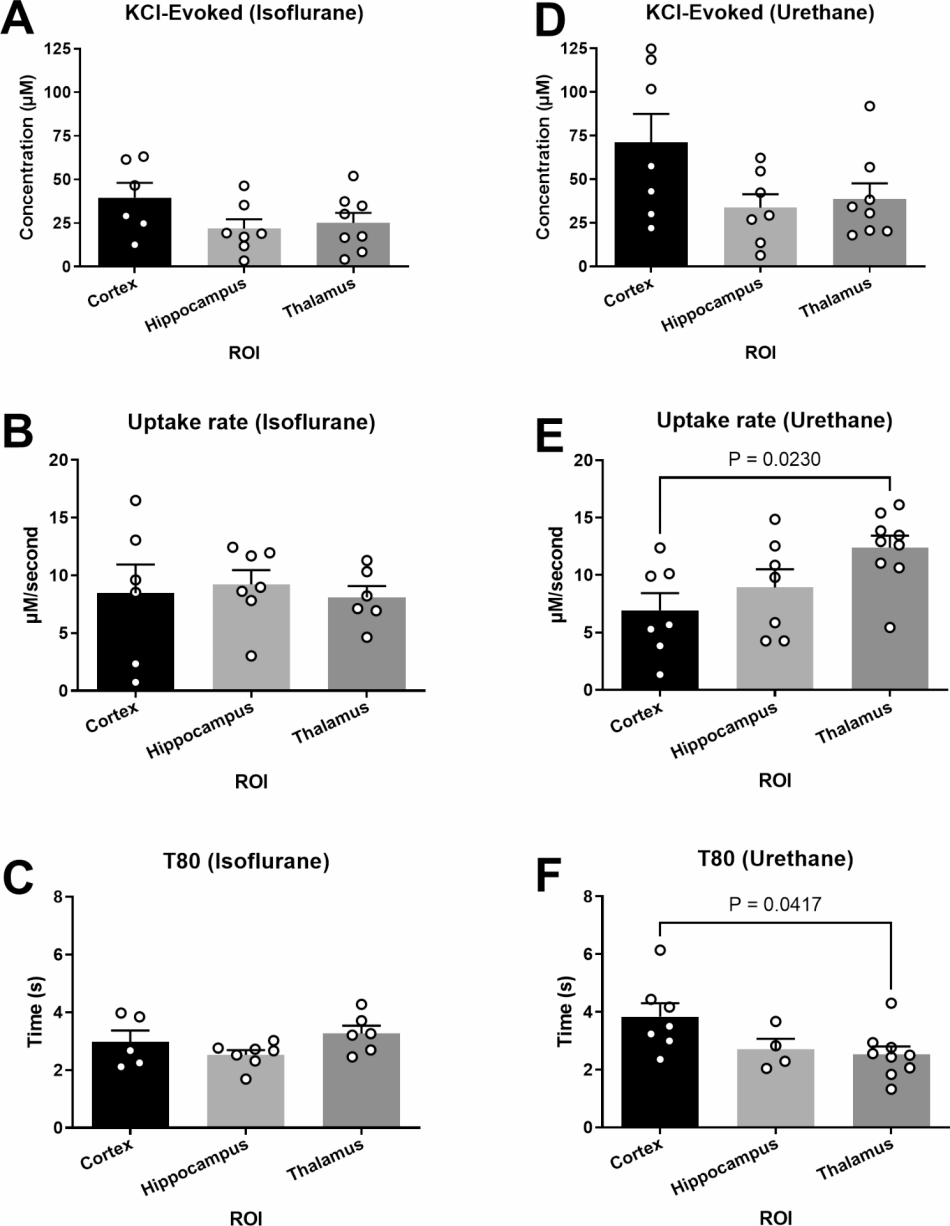
**Glutamate clearance under urethane was capable of distinguishing region-dependent differences**. (**A**-**C**) Under isoflurane, all outcome measures were similar between the cortex, hippocampus, and thalamus. (**D**) No significant differences were detected in KCl-evoked glutamate release across regions when using urethane. (**E**-**F**) Glutamate clearance kinetics under urethane changes as a function of the brain region when evaluating uptake rate (F_2,20_ = 4.41; p = 0.026) and T_80_ (F_2,14_ = 3.79; p = 0.044), where uptake rate was significantly faster and clearance time was significantly shorter in the thalamus compared to the cortex. Bar graphs represent mean + SEM. *N* = 6–9 per group

Aspects of glutamate neurotransmission were compared between brain regions under the influence of single anesthetic to determine region-specific differences. No significant differences were detected between the cortex, hippocampus, and thalamus when using isoflurane for KCl-evoked glutamate overflow, uptake rate, and T_80_ (Fig. 7A-C). Additionally, no differences were detected between regions for KCl-evoked overflow (Fig. 7D) when using urethane anesthesia. Region-dependent differences were detected in glutamate clearance, with both the uptake rate (F_2,20_ = 4.41; p = 0.026; Fig. 7E) and T_80_ (F_2,14_ = 3.79; p = 0.044; Fig. 7F) being statistically significant between the thalamus and cortex recordings. Uptake rate in the thalamus was significantly faster than the cortex (p = 0.023), with no differences detected between the cortex or thalamus and the hippocampus ($\stackrel{-}{\text{x}}$_Ctx−Ure_ = 6.938 µM/sec, $\stackrel{-}{\text{x}}$_Hippo−Ure_ = 8.923 µM/sec, $\stackrel{-}{\text{x}}$_Thal−Ure_ = 12.40 µM/sec). Faster uptake rate in the thalamus was supported by a shorter time to clear 80% of the glutamate signal in comparison to the cortex ($\stackrel{-}{\text{x}}$_Ctx−Ure_ = 3.834 s, $\stackrel{-}{\text{x}}$_Hippo−Ure_ = 2.708 s, $\stackrel{-}{\text{x}}$_Thal−Ure_ 2.525 s; p = 0.042).

## Discussion

These experiments were designed to test whether KCl-evoked glutamate overflow and glutamate clearance kinetics in the cortex, hippocampus, and thalamus were similar in rats anesthetized with either urethane or isoflurane. No differences in KCl-evoked glutamate overflow were identified by evaluating the maximal amplitude of glutamate released. The uptake rate and clearance time (T_80_) of locally applied exogenous glutamate were also similar under both anesthetics in the cortex and hippocampus; however, the uptake rate under urethane was significantly faster in the thalamus compared to isoflurane. Further, urethane treatment allowed the capability of measuring region-specific differences, where all regions were similar with isoflurane anesthetization. These data support the need for careful interpretation when comparing recordings from rodents under urethane or isoflurane anesthesia, as certain parameters of glutamate uptake vary based on the choice of anesthesia and brain region.

Local application of KCl causes a net positive of the resting membrane potential of surrounding neurons and glia, which results in subsequent action potentials that release stored neurotransmitters. The depolarization through KCl is thought to largely reflect synaptic release while Ca^2+^-dependent vesicular release by glia also occurs [37–40]. The glutamate-selective MEAs record sub-second changes in glutamate overflow into the extracellular space, such that the maximum amplitude can be statistically evaluated. Alterations of values obtained from KCl-evoked glutamate overflow may result from changes to presynaptic neuron release, glutamate output from surrounding glia, decreased glutamate clearance, and the changes to glutamate transporters, GABAergic or modulatory neurotransmission, or alterations to mGluR regulation of glutamate release [18, 28, 41–43]. A larger glutamate release in response to KCl-evoked depolarization closely resembles excessive excitability as previously described [44], which would indicate circuit excitability. In these experiments, no difference in evoked glutamate release was determined as a function of anesthesia or region. Both urethane and isoflurane can dampen evoked responses, where urethane has been shown influence physiological evoked responses less than isoflurane, hypothesized to be due to isoflurane increasing tonic GABAergic inhibition [17, 45]. However, our data suggest that KCl-evoked glutamate levels are similar when the depth of anesthesia results in loss of reflexes and rapid, shallow, breathing recommended for surgical procedures. Perhaps, the strong depolarization of 120 mM KCl overpowers any inhibitory effects of isoflurane, where using smaller volumes or lower concentrations of KCl may be able to detect anesthesia-dependent differences.

Local application of exogenous glutamate enables the study of glutamate clearance kinetics in the extracellular space. The main contributors to the clearance of glutamate from the extracellular space in the cortex, hippocampus, and thalamus are astrocytic glutamate transporters EAAT1 (GLT-1; cerebral cortex: 90%; thalamus: 54%; data in comparison to the hippocampus) and EAAT2 (GLAST; cerebral cortex: 33%; hippocampus: 35%; thalamus: 22%; data in contrast to the cerebellum), and to a lesser extent, post-synaptic transporters EAAT3/4 [1, 46, 47]. GLT1 (EAAT2) is located on glia and is responsible for 90% of the glutamate uptake. GLAST (EAAT1) is also predominantly on glia, where GLT1 is typically 4x GLAST with the exception of Bergmann glia in cerebellum [1, 48–50]. Changes in glutamate clearance may indicate the function of glutamate transporters, transporter trafficking, transporter capacity, and transporter affinity [51]. Extracellular clearance parameters, including uptake rate, represent the velocity of the transporters, while T_80_ indicates the affinity of glutamate to the transporters [52]. Previous work has shown that alterations to the location (trafficking) or expression of GLT-1 and GLAST correlate to changes in glutamate clearance parameters as well [53, 54]. Conflicting literature evaluating the influence of isoflurane on glutamate uptake in in vitro models indicates enhancements in a dose-dependent manner [55–57], suppression/inhibition [58, 59], or no change [60]. Further, isoflurane use has been shown to increase the surface-level expression of EAAT3 [61].

While studies addressing the influence of urethane on glutamate transporters are lacking, the present data indicate that urethane interacts with the neurotransmitter system in the thalamus differently, such that regional-dependent differences in glutamate clearance can be detected. Borrowing data from our previous finding [18], where we used a similar experimental paradigm under isoflurane anesthesia, we compared the uptake rate between the cortex and the thalamus and did not detect a significant difference using a two-tailed Student’s t-test (t_16_ = 0.38; p = 0.71). Similarly, borrowing data from Krishna et al., 2020, where we replicated these experiments under urethane anesthesia, we compared the update rate constant between the cortex and the thalamus, where a higher uptake rate constant in the thalamus approached significance compared to cortex (t_17_ = 1.89; p = 0.077) [23]. The primary difference between the experimental design in our previous publications and the present study is the inclusion criterion for amplitude matching, where previously we analyzed amplitudes from ~ 7–15 µM and these experiments included 10–23 µM, where the overall larger amplitude size could have emphasized region-dependent differences. Thus, overall, our previous publications support the reported region-dependent differences. Perhaps cellular, laminar, and regional heterogeneities of glutamate transporter distribution in the thalamus are contributing to this effect [62]. Subpopulation of the thalamic astrocytes expresses AMPA receptors that are not present in other brain regions, indicating unique characteristics of the thalamus across multiple brain regions [63], which may be evolutionarily conserved due to the thalamus’s fundamental demands for information processing and integration. Additional studies are needed to confirm whether this effect is due to the anesthetics having a differential influence on transporter function/expression or whether other distinct mechanisms of action culminate in minor region-dependent clearance kinetics.

The pharmacological mechanisms for anesthesia by urethane and isoflurane need to be better understood at the neurochemical level, making it difficult to predict the effect on aspects of neurotransmission [64]. Both urethane and isoflurane have been indicated to dampen overall glutamate neurotransmission, reported by evidence of less glutamate released, altered reuptake, reduced cellular excitability, decreased evoked glutamate overflow, and decreased tonic levels [7, 58, 65–70]. These effects may be mediated through the interaction between urethane and isoflurane with various GABAergic and glutamatergic receptors, as well as similar antagonistic effects at NMDA and AMPA receptors [71, 72]. Local field potential values were also similar under both anesthetics [64]. While each respective anesthetic’s molecular mechanism remains only partially understood, similarities amongst molecular targets and aspects of neuronal communication may contribute to comparable alterations in glutamate neurotransmission. Alternate sites of action of the anesthetics indicate a dose-dependent influence on factors that mediate glutamate neurotransmission [71, 73]. Isoflurane has been shown to bind to GABA_a_ receptors [74], glutamate receptors, and glycine receptors, inhibit potassium channels [75, 76], and hyperpolarize neurons [77]. Hara et al. describes several unique molecular targets of urethane that influence glutamate neurotransmission, including enhancement of GABA_a_ and glycine receptors, while various NMDA and AMPA subtypes are inhibited dose-dependently [71]. Furthermore, a 30% decrease in glutamate concentrations in the cerebral cortex of rats under urethane exposure is observed compared to unanesthetized rats [7]. It is indicated that urethane and isoflurane suppress presynaptic glutamate release associated with increased excitatory post-synaptic currents (EPSCs) in the hippocampus and thalamus, respectively [8, 78]. Further, it is shown that glutamate neurotransmission is reduced in cortical inhibitory interneurons with urethane exposure [79]. These reports indicate that other parameters, like basal extracellular levels, electrophysiological characteristics, and cell types not evaluated in these experiments, could be disrupted. Importantly, a recent study indicated that both isoflurane and urethane exposure had no significant impact on the neuronal response to whisker stimulation in mice [18, 23, 80, 81].

Anesthesia offers a valuable tool for neurochemical analysis, whether via amperometry, microdialysis, electrophysiology, or other methods. Such analysis allows for examining neuronal communication across multiple regions and detecting pathological shifts compared to controls within relevant circuits [82]. However, it’s essential to note that animal-specific factors, such as species, strain, age, and sex, impact glutamate neurotransmission. The mechanisms behind these influences remain largely theoretical and might obscure true biological relevance [18, 23, 53, 83–88]. Our data indicate that glutamate clearance parameters did not significantly differ between isoflurane and urethane in the cortex and hippocampus, despite being accomplished through dissimilar mechanisms. Yet, reports on the broader implications of these anesthetics on neuronal communication are more varied. For example, isoflurane has been shown to dampen sub-cortical activity, inducing synchronous cortico-striatal fluctuations, while using urethane resulted in recordings more similar to awake rodents [10]. Neuronal activity measured utilizing BOLD fMRI reported that both isoflurane and urethane dampens responses when compared to unanesthetized subjects but does not comment on which anesthetic is more comparable to the awake state [89–91]. Studies investigating changes in molecular components that are known to interact with a given anesthetic must use caution when interpreting results and attributing changes to a specific mechanism. These and other studies indicate that not all neuronal circuits respond identically under anesthesia. Thus, investigating anesthesia’s effect on other neuromodulators (5-HT, GABA, DA, ACh) and their functional outputs in specific circuits should be considered when interpreting comparisons across several studies, despite PCO_2,_ O_2_, pH, and heart rate being similarly affected [10]. Therefore, experiments should be carried out in preparation for moving into awake-behaving models, where simultaneous recordings of behavior and glutamate signaling are possible. In doing so, it is possible to measure behaviorally relevant aberrant glutamate signaling and determine the efficacy of therapeutic approaches to treat several brain disorders marked by maladaptive behaviors.

The safety and efficacy of anesthetic on animals has historically influenced researchers’ choice when considering study design. Urethane is classified by the International Agency for Research on Cancer as a group 2 A carcinogen and thus considered “probably carcinogenic to humans.“ This designation has limited urethane use primarily to non-survival studies despite its ease of use and favorable profile. In doing so, using urethane as a sole anesthetic in longitudinal studies with multiple data collection time points in the same animals is not considered humane for the risk of tumor formation. However, the inclusion of urethane as part of an anesthetic cocktail provides researchers with significant advantages. In mice, it was found that using 560 mg/kg alongside ketamine and xylazine was not carcinogenic [92]. This mixture proves beneficial, as lower doses of all three anesthetics can minimize the confounding alterations in electrical potentials often seen when a ketamine/xylazine duo is administered in isolation [92–94]. Importantly, these studies underscore the potential advantages of combining various anesthetics to harness their individual strengths.

A limitation of these experiments is that they were conducted exclusively in male subjects, while recent evidence has shown that glutamate clearance differs between males and females, with females exhibiting slower clearance in certain brain regions [23]. Considering the NIH’s mandate for the inclusion of females in research and the absence of comparable foundational data, the risk to data integrity, interpretation, rigor, and reproducibility is magnified. Future studies must address this limitation by including females to ensure a comprehensive understanding of glutamate dynamics and its potential sex-specific differences.

## Conclusions

Glutamate signaling is essential for synaptic plasticity and associated behaviors in healthy and pathological states [95, 96]. Understanding glutamate neurotransmission in limbic brain regions, crucial for learning, sensory, and affective/ anxiety-like behaviors, can serve as a marker for both functional and dysfunctional brain activity. However, the utilization of anesthetics in animal research can introduce variables that influence glutamate neurotransmission, thereby adding complexity to data interpretation. Our study uniquely investigates in vivo glutamate dynamics under urethane and isoflurane anesthesia, revealing similar KCl-evoked glutamate overflow but distinct clearance parameters in different brain regions. When interpreting electrochemical recordings under either anesthetic, researchers should consider other potential influencing factors, particularly the region of interest. Future studies should optimize experimental designs, capitalizing on the inherent advantages of chosen anesthetics, and include females to evaluate potential sex-specific sensitivities. In summary, our results accentuate the need for meticulous experimental design and region-specific consideration when examining glutamate dynamics in the backdrop of anesthesia.

## Data Availability

All data that support the findings of this study are available from the corresponding author upon request.

## List of Abbreviations

AD: Alzheimer’s disease
ADHD: Attention-deficit/hyperactivity disorder
CNS: Central nervous system
HD: Huntington’s disease
KCl: Potassium chloride
LOD: Limit of detection
MEAs: Microelectrode arrays
PBS: Phosphate buffered saline
PD: Parkinson’s disease
PFA: Paraformaldehyde
ROI: Region of interest
